# When drought meets heat – a plant omics perspective

**DOI:** 10.3389/fpls.2023.1250878

**Published:** 2023-08-22

**Authors:** Xiangyu Xu, Cassio Flavio Fonseca de Lima, Lam Dai Vu, Ive De Smet

**Affiliations:** ^1^ Department of Plant Biotechnology and Bioinformatics, Ghent University, Ghent, Belgium; ^2^ VIB Center for Plant Systems Biology, Ghent, Belgium

**Keywords:** drought, heat, transcriptomics, proteomics, metabolomics

## Abstract

Changes in weather patterns with emerging drought risks and rising global temperature are widespread and negatively affect crop growth and productivity. In nature, plants are simultaneously exposed to multiple biotic and abiotic stresses, but most studies focus on individual stress conditions. However, the simultaneous occurrence of different stresses impacts plant growth and development differently than a single stress. Plants sense the different stress combinations in the same or in different tissues, which could induce specific systemic signalling and acclimation responses; impacting different stress-responsive transcripts, protein abundance and modifications, and metabolites. This mini-review focuses on the combination of drought and heat, two abiotic stress conditions that often occur together. Recent omics studies indicate common or independent regulators involved in heat or drought stress responses. Here, we summarize the current research results, highlight gaps in our knowledge, and flag potential future focus areas.

## Introduction

**Figure 1:**
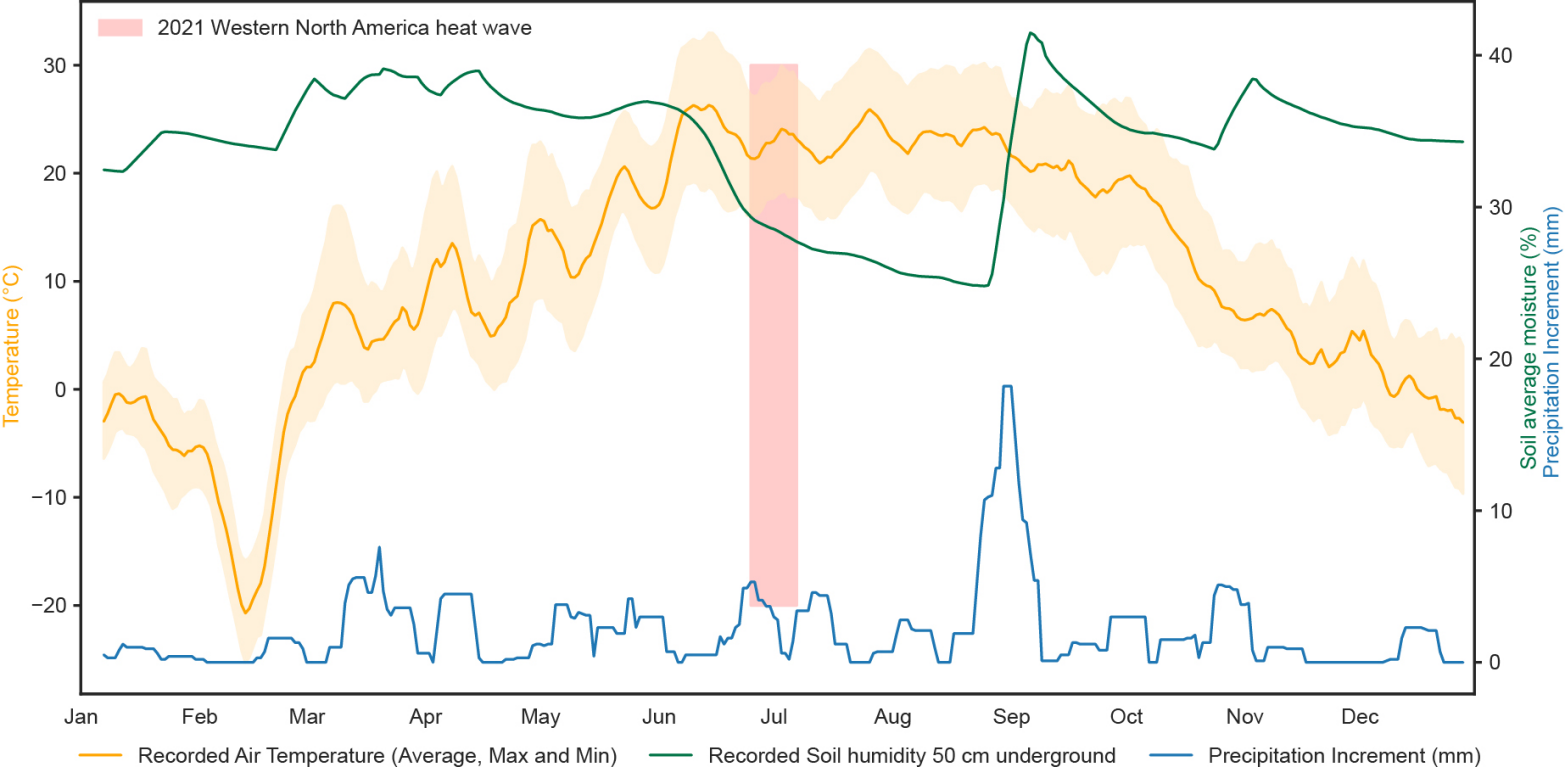
Representative meteorological data of high temperature and water availability stress co-occurring in field conditions from the Shagbark Hills (2068) station in Iowa - USA, sourced from SCAN (Soil Climate Analysis Network) (USDA Natural Resources Conservation Service, 2022). The 10-day moving average of air temperature anomalies (orange), soil humidity (%) at 50 cm depth (green) and precipitation increments (blue) were plotted for the year 2021 in the location. Soil average moisture remained stable along the seasonal fluctuations of air temperature, but sharply decreased after the temperature peaked and the 2021 Western North America heat wave (25/06/2021 - 07/07/2021) took place (highlighted in red) (Wikipedia contributors, 2023). The soil moisture level was only restored to its previous level around late August. The data suggest that, in the surrounding crop field areas, plants were exposed to moderate/high temperature stress prior to the exposure to drought.

Plants are sessile organisms that cannot escape from adverse conditions, and are thus at the mercy of biotic and abiotic environmental factors that strongly affect their growth, survival and performance (Suzuki et al., 2014; Zhang et al., 2022). Furthermore, climate change, especially the change of the limiting factors temperature and water availability, vastly reduces crop yields, which threatens productivity and ultimately food security (Bailey-Serres et al., 2019). These different environmental stresses can be perceived in the same or in different tissues with specific systemic signalling and acclimation responses. For example, plants generally recognize drought stress in the soil through the root system and transmit a signal to the shoot (Takahashi et al., 2018; Maurel and Nacry, 2020), while high temperature stress is predominantly perceived in the aboveground parts (Bita and Gerats, 2013; Hasanuzzaman et al., 2013; Quint et al., 2016). The response of plants to individual heat or drought stress and the underlying specific signalling pathways are well-studied (Quint et al., 2016; Vu et al., 2019b; Gupta et al., 2020), but in several cases these stresses coincide (**Figure 1**) and thus likely impact plants differently than the individual stresses (Suzuki et al., 2014; Zandalinas et al., 2020a; Zandalinas et al., 2020b). When exposed to combined stressors, different types of interactions, such as additive, synergistic, equalization, dominant, and antagonistic effects can occur, leading to the induction of diverse stress-responsive transcripts, proteins, and metabolites (Shaar-Moshe et al., 2017). Omics studies provide a holistic view on these change and can uncover complex regulatory pathways in which a large number of transcripts, proteins, and metabolites undergo similar or opposite changes, highlighting differences between combined and individual stresses.

Here, we will discuss recent findings related to combined drought and heat stress (referred to as combined stress). We will focus on how plants adapt to this stress combination through developmental and physiological processes and how different omics levels are regulated. Since the majority of omics data is on the aboveground parts of the plants, we mainly describe these (unless stated otherwise) (**Supplementary Table 1**).

## Developmental and physiological responses to individual and combined heat and drought stress

As air temperature rises, the water content in soil tends to decrease, indicating that temperature and water availability stress are likely to co-occur in field conditions (Lobell and Gourdji, 2012; Konapala et al., 2020; Bevacqua et al., 2022; Coughlan de Perez et al., 2023). This outcome of probabilistic meteorological models that highlight the frequency of combined heat and drought events can already be observed in the field (**Figure 1**). Both drought and heat stress individually influence seed germination, cell division and expansion, photosynthesis, and yield (Avramova et al., 2015; Quint et al., 2016; Nelissen et al., 2018; Vu et al., 2019b; Gupta et al., 2020; Tiwari et al., 2022). Depending on the severity (water content in the soil) and the duration, drought stress can vary considerably. Mild drought stress slows down growth, resulting in a decrease in leaf area and reduction in biomass, and reduces yield (Verelst et al., 2013; Clauw et al., 2015). Severe drought stress has a far more devastating impact on plant physiology, causing growth to nearly cease, plants to wilt and ultimately resulting in plant death (Harb et al., 2010; Muller et al., 2011). Drought also leads to stomatal closure to reduce evaporation as a rapid defence against dehydration (Buckley, 2019; Gupta et al., 2020). Similarly, the impact of temperature stress also depends on the frequency, severity and duration of the stress (Zhu et al., 2022). Exposure to a mildly increased ambient temperature can induce various alterations in plant architecture to move sensitive parts away from high temperature and improve cooling capacity and trigger floral transition (Quint et al., 2016; Vu et al., 2019b). A further increased temperature and a high frequency and/or prolonged duration of high temperature can decrease germination rates, inhibit growth and floral transition, result in a reduction in yield and even lead to plant death (Gan et al., 2014; Chiu et al., 2016; Quint et al., 2016; Wu et al., 2017; Li et al., 2019; Vu et al., 2019b; Li et al., 2020; Zhu et al., 2021; Ying et al., 2022; Zhu et al., 2022). Elevated temperatures can have a positive effect on the photosynthetic rate and carbon assimilation in plants, but this beneficial effect is strongly suppressed once a certain threshold temperature is exceeded (Yamasaki et al., 2002; Shaar-Moshe et al., 2017; Yang et al., 2020). Finally, high temperatures lead to stomatal opening and an increased stomatal conductance associated with leaf cooling, and prolonged exposure to high temperature reduces the stomata number (Yamori et al., 2006; Crawford et al., 2012).

The simultaneous occurrence of high temperature and drought stress can further suppress plant growth and yield compared to their individual effects. When Arabidopsis and different food crops are exposed to combined stress, stems are shorter and leaves are less abundant, the fresh weight and viability of pollen are further decreased, the seed yield and fresh weight are also further decreased compared to control plants and/or to individual stress conditions (Mishra et al., 2018; Choukri et al., 2020; Demirel et al., 2020; Cohen et al., 2021; Lehretz et al., 2021; Li et al., 2022; Mahalingam et al., 2022; Rahman et al., 2022). In contrast, under combined stress, the transpiration response in Arabidopsis is dominantly promoted by heat stress compared to the individual stress, whereas in the individual stress, it is promoted by high temperature but repressed by drought stress compared with normal conditions (Rizhsky et al., 2004). In Arabidopsis and soybean, heat and drought also antagonistically regulate stomatal movement, but in this context drought dominantly decreases stomatal conductance and photosynthesis in combined heat and drought stress conditions (Rizhsky et al., 2004; Sinha et al., 2022).

To identify the molecular machinery involved in plant regulation and acclimation under combined stress conditions, a comprehensive analysis of the transcriptome, proteome, post-translational modifications (PTMs) and metabolome is essential.

## Transcriptome responses to combined heat and drought

In Arabidopsis, drought stress significantly impacts gene expression in plants, primarily of genes associated with hormone-mediated growth regulation, response to osmotic stress, reactive oxygen species, salt stress, cell wall modification and cell growth (Clauw et al., 2015). High temperature predominantly induces an up-regulated transcriptional response to heat, protein folding and metabolic process in Arabidopsis (He et al., 2019). A 7-day individual heat or drought treatment leads to more differentially expressed genes (DEGs) compared to a 3-day treatment under individual stress in the barley flag leaf (Mikołajczak et al., 2023).

**Figure 2:**
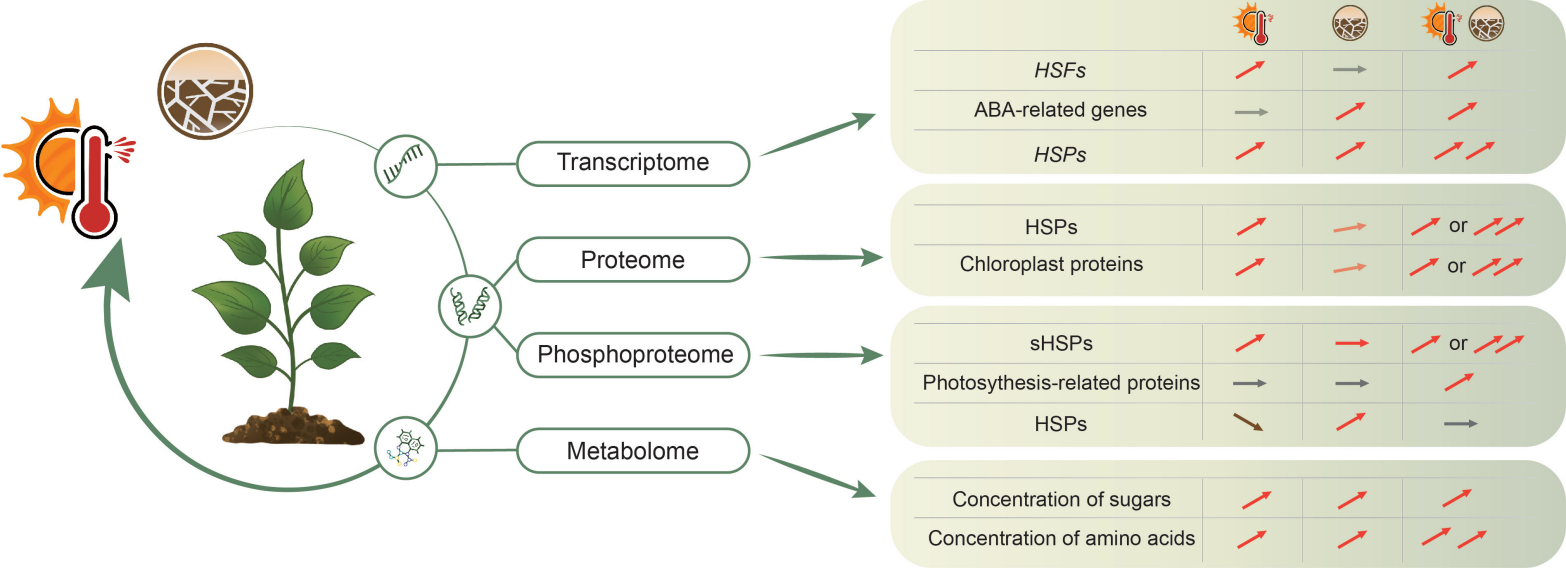
Combined heat and drought stress can differentially regulate plant transcriptome, proteome and PTMs (post-translational modification), and metabolome in plants to adapt to environmental changes. Different omics reveal varying regulatory patterns for several regulators under conditions of individual heat and drought or combined heat and drought stress. HSFs, Heat Shock Transcription Factors; HSPs, HEAT SHOCK PROTEINs; sHSPs, small HEAT SHOCK PROTEINs. The icon with the thermometer and sun indicates the heat stress and the cracked land indicates the drought stress. The arrows indicate increase, decrease or no change under indicated conditions.

The biological processes associated with responses to a chemical, a stimulus, an oxygen-containing compound, and to stress are all highly enriched in drought, heat and combined stress treatments in lentil leaves (Hosseini et al., 2021), indicating overlapping signalling pathways. Combined stress induces differentially expressed genes in food crops associated with the ribosome pathway and with photosynthesis and chloroplast-related processes compared with control conditions, and uniquely up- and down-regulated genes enriched in metabolic and biosynthetic processes of the organonitrogen compound, peptide and amide, translation and cytoplasm-related terms (Hosseini et al., 2021; Tan et al., 2023). Restructuring of the transcriptome due to the simultaneous occurrence of heat and drought stress varies in different studies. The transcriptomic signature, such as the percentage of overlapping DEGs, indicates that either high temperature or drought plays a major regulatory role in the transcriptome under combined stress, such as the expression patterns of most *HEAT SHOCK TRANSCRIPTION FACTORS (HSFs)* are predominantly regulated by heat, while of most abscisic acid (ABA)-related genes are primarily regulated by drought (Rizhsky et al., 2004; Shaar-Moshe et al., 2017; Sinha et al., 2022; Mikołajczak et al., 2023; Sinha et al., 2023) (**Figure 2**). In addition, additive/synergistic transcriptional responses to combined stresses, often with a dominant impact of one stress, also occurs. For example, the expression of some *HEAT SHOCK PROTEINs* (*HSPs*) quickly responds to individual high temperature or drought stress with a significant increase, and the combination of high temperature and drought additionally increases their expression (Rizhsky et al., 2002; Rizhsky et al., 2004; Mahalingam et al., 2022; Rahman et al., 2022) (**Figure 2**). However, plants subjected to combined stress, also display different DEG response patterns compared to individual heat or drought stress, indicating a limited expression overlap among individual stresses and combined stresses (Rizhsky et al., 2004; Liu et al., 2018; Hosseini et al., 2021; Mikołajczak et al., 2023; Sinha et al., 2023; Tan et al., 2023). For example in wheat, combined stress induces specific alternative splicing that is absent under individual stresses, and some of these alternatively spliced genes are associated with glutathione biosynthesis and DNA methylation (Liu et al., 2018). Furthermore, under combined stress, a significant number of transcripts are oppositely regulated compared to each individual stress, or distinct from the effect of the individual stress showing expression levels of untreated plants (Rizhsky et al., 2004; Sinha et al., 2023). However, among these unique transcripts induced by combined stress, a much lower overall similarity was found in different soybean organs (Sinha et al., 2023), indicating a unique transcriptional response in different plant organs, and emphasizing the importance of focused studies to understand tissue and organ-specific responses to combined stress conditions.

These different types of interactions of DEGs detected under combined stress occur in different processes and pathways, which are largely related to photosynthesis and encoding mitochondrial proteins (Rizhsky et al., 2004; Hosseini et al., 2021). GO terms associated with these unique DEGs also pinpoint the response to ABA and the metabolic and biosynthetic processes of organonitrogen (Rizhsky et al., 2004; Hosseini et al., 2021; Mikołajczak et al., 2023).

## Proteome responses to combined heat and drought

Changes in gene expression will – to some extent – result in changes at the protein level (Greenbaum et al., 2003); and regulation of translation, protein abundance and protein activity through, for example, post-translational modifications, add another layer of regulation to the proteome.

Under drought conditions, there was a significant induction in the abundance of proteins in maize related to carbohydrate metabolism pathways, including glycolysis and the pentose phosphate pathway (Vu et al., 2016). Conversely, proteins involved in chromatin organization, including several histones, exhibited an overall decrease in expression levels (Vu et al., 2016). High-temperature stress significantly affects protein structure and stability in Arabidopsis, especially those involved in ribosomal proteins/nucleic acid binding, proteasomal proteins, and cytoskeletal proteins (Volkening et al., 2019). These affected processes of differentially expressed proteins (DEPs) also depend on the species and on the developmental stage (Liu et al., 2015; Lu et al., 2017; Zhang et al., 2017; Liu et al., 2021).

Based on a limited number of studies, the abundance of the differentially regulated crop proteins under combined stress is primarily associated with ribosomes, metabolic processes and photosynthesis (Rollins et al., 2013; Zhao et al., 2016; Tang et al., 2023). Combined stress, and either one or both individual stress conditions, share a large proportion of DEPs of enzymes in maize leaves, such as kinases, phosphatases, enzymes involved in phytohormone signalling, or other metabolic enzymes (Zhao et al., 2016). Some DEPs are predominantly regulated by a single stress and/or exhibit additional regulation under combined stress. For example, the abundance of the majority of identified HSPs or chloroplast proteins in sweet potatoes is up-regulated by heat stress and only slightly affected by drought stress, and under combined stress, an additional increase is observed (Tang et al., 2023) (**Figure 2**).

Post-translational modifications (PTMs) increase the functional diversity of the overall proteome. While several studies have explored PTMs under single drought or heat stress conditions (Scharf and Nover, 1982; Castro et al., 2012; Zhang et al., 2014; Vu et al., 2016; Botha et al., 2017; Chen et al., 2017; Benlloch and Lois, 2018; Vu et al., 2018; Morrell and Sadanandom, 2019; Xu and Xue, 2019; Pengyan et al., 2020; Zhang et al., 2020; Han et al., 2021; Han et al., 2022), there are only a few studies that have investigated phosphorylation, under combined stress conditions. Under drought conditions, the pathways primarily associated with sodium transport, immune response, and chromatin silencing of detected phosphorylated protein are affected in maize leaves (Vu et al., 2016). Differentially phosphorylated proteins upon mild heat in wheat leaves, compared to non-stress conditions, are enriched in biological processes associated with heat, protein folding, response to hydrogen peroxide and glucose transport (Vu et al., 2018). Combined stress differentially regulates protein phosphorylation in the maize leaf, and out of 282 phosphoproteins, 46 of them are common between individual stress and combined stress (Hu et al., 2015). The phosphorylation level of detected HSPs and small HSPs (sHSPs) in maize is mainly regulated in response to heat and combined stress, but does not significantly change under drought (Hu et al., 2015) (**Figure 2**). However, several phosphoproteins related to photosynthesis, carbon metabolism and protein processing are detected under combined high temperature and drought conditions (Hu et al., 2015) (**Figure 2**).

Other common PTMs, such as ubiquitination and sumoylation, targeting Lys residues, have also been studied under individual heat and drought conditions (Catala et al., 2007; Miura et al., 2009; Castro et al., 2012; Li et al., 2015; Wu et al., 2016; Xu and Xue, 2019; Pengyan et al., 2020; Andrási et al., 2021; Han et al., 2021). However, there is limited available data on the ubiquitinome and sumoylome in the context of combined stress. Nevertheless, high temperature or drought-induced ubiquitination regulates ABA signalling (Chiu et al., 2016; Yu et al., 2016; Xu and Xue, 2019), and ubiquitination regulates drought tolerance via the ABA signalling pathway (Seo et al., 2012; Lim et al., 2017; Xu and Xue, 2019; Tong et al., 2021; Singh et al., 2022). High temperatures inhibit Arabidopsis seed germination by dampening both protein ubiquitination and proteasome activity in an ABA-dependent manner (Chiu et al., 2016). The differently ubiquitinated proteins under high temperatures are enriched in a wide range of molecular functions (Li et al., 2015; Pengyan et al., 2020). Sumoylation can be stimulated in plants under heat (Miller et al., 2010; Miller et al., 2013; Hendriks and Vertegaal, 2016; Rytz et al., 2018; Han et al., 2021) or drought stress (Catala et al., 2007; Miura et al., 2009; Castro et al., 2012; Wu et al., 2016; Joo et al., 2022) and largely depends on SUMO E3 ligase SAP AND MIZ1 DOMAIN- CONTAINING LIGASE 1 (SIZ1). SIZ1 facilitates conjugation of SUMO to protein substrates, and positively regulates plant heat tolerance and acquired thermotolerance (Yoo et al., 2006; Kim et al., 2017; Rytz et al., 2018). Sumoylation occurring on chromatin is associated with gene expression in response to high temperature (Niskanen et al., 2015; Han et al., 2021). The transcripts that are differentially regulated by sumoylation are largely involved in responses to heat stress and development-related processes (Han et al., 2021). Some studies exhibit that SIZ1 has both positive and negative effects on drought tolerance (Catala et al., 2007; Miura et al., 2009; Vu et al., 2016; Benlloch and Lois, 2018; Xu and Xue, 2019). So far as we know, there are no omics data on sumoylation under drought or combined stress conditions, but overexpression of SIZ1 enhances photosynthesis performance and yield compared to the wild type under combined stress (Mishra et al., 2017). This suggests a complex regulation of sumoylation via SIZ1, which might be affected by the stress intensity and potentially plays an important role under drought or combined heat and drought stress.

## Metabolome responses to combined heat and drought

Under combined stress, plants may experience a reduction in growth and yield as mentioned above. Under such conditions, the levels of biological markers of oxidative stress, such as malondialdehyde (MDA) and H_2_O_2_, further increase (Jin et al., 2016; Rahman et al., 2022).

To safeguard cells from stress-induced damage, plants adapt by reprogramming their metabolic pathways. While there are several metabolome datasets under individual drought and heat stress (Ye et al., 2016; Sun et al., 2019; Abdelrahman et al., 2020; Lecourieux et al., 2020; Guo et al., 2021; Lu et al., 2022; López et al., 2023; Xie et al., 2023), there are only a few studies with respect to combined stress (Zinta et al., 2018; Alhaithloul et al., 2019; Lawas et al., 2019; Qaseem et al., 2019; Xie et al., 2020; Yousaf et al., 2022; Ru et al., 2023). Under combined stress, the processes of carbohydrate metabolism, amino acid metabolism and organic acid are differentially affected compared with a non-stress condition (Zinta et al., 2018; Lawas et al., 2019). There is an increase in stress-responsive metabolites in flowering spikelets, particularly in terms of their abundance under severe combined heat and drought stress, compared to mild combined stress conditions (Lawas et al., 2019). Under combined stress, there is a significant and strong transient increase in the concentration of most soluble sugars, such as glucose, fructose, and raffinose (**Figure 2**), although the concentration of sucrose and starch decreased compared to a non-stress condition (Zinta et al., 2018; Alhaithloul et al., 2019). These soluble sugars increase the osmotic potential in the cell, drawing water into these cells to maintain the turgor pressure (Fàbregas and Fernie, 2019), and act as protectants to cope with rapid stress (Rosa et al., 2009; Du et al., 2019). Under combined stress, some results show that the total amount of soluble sugar further increased compared to individual heat and drought stress (Qaseem et al., 2019; Ru et al., 2023). However, this increased effect of soluble sugar exhibits variation across different varieties and species, in response to combined stress compared with individual stress or control conditions, providing a possible explanation for the variable tolerance observed among different varieties and species (Zhou et al., 2017; Qaseem et al., 2019; Alsamadany et al., 2023; Ru et al., 2023).

The concentrations of amino acids exhibit a distinct profile when subjected to combined stress (Zinta et al., 2018). Under combined stress, a strong transient increase of amino acids, such as histidine, isoleucine, leucine, methionine, and proline, are observed (**Figure 2**), while alanine, asparagine, and aspartate do not show a difference under these combined treatments (Zinta et al., 2018; Lawas et al., 2019; Xie et al., 2020; Yousaf et al., 2022).

The concentration of fatty acids is also differentially impacted by combined stress compared with a non-stress condition, and both saturated (SFA) and unsaturated fatty acids (UFA) exhibit specific temporal patterns. The concentration of mainly SFAs increases during exposure to stress (**Figure 2**), while mono- and poly-UFAs mostly decrease or remain unchanged during stress (Zinta et al., 2018). The increase in SFAs and decrease in UFAs following prolonged stress could potentially be linked to the adaptation of membranes in managing fluctuations in fluidity. The membrane plays an important role in signal perception and transduction (Inda et al., 2014; Niu and Xiang, 2018). For example, high temperature promotes membrane fluidization, while hyperosmotic stress can reduce membrane fluidity (Laroche et al., 2001; Los and Murata, 2004; Mansilla et al., 2004; Martinière et al., 2011; Leach and Cowen, 2014; Vu et al., 2019a). The pH value surrounding the cell membrane can affect its permeability and polarity, and drought stress can trigger cytoplasmic alkalinisation, thereby impacting membrane dynamics (Geilfus, 2017; Angelova et al., 2018). The different modifications of signal perception under combined stresses might lead the distinct signal transduction, which can also influence the activity of membrane-associated proteins and downstream targets (Niu and Xiang, 2018; Vu et al., 2019a).

## Conclusion

Omics experiments under combined heat or/and drought stress, provide systems level knowledge on how plant growth and yield, and the associated physiological and biochemical responses, are regulated under stress (**Figure 2**). Heat and drought stress may exert distinct effects on various tissues or organs. However, due to the limited number of omics datasets, and the majority of studies being conducted on leaf or whole plants, the potential interplay between organs and signalling pathways remains largely unexplored. Although the initial sensing of these stresses likely occurs locally and specifically, the resulting biochemical signals can be quickly transferred to and perceived by other tissues and organs. This coordinated regulation between local and transferred signals might explain the different regulations observed under combined stress that are absent under a single stress. There are several conserved responses to both heat and drought stress, including those related to ABA signalling and heat shock proteins (Rizhsky et al., 2002; Rizhsky et al., 2004; Zhao et al., 2016; Marín-de la Rosa et al., 2019; Andrási et al., 2021; Tamang et al., 2021; Wang et al., 2021; Mahalingam et al., 2022; Tang et al., 2023).

Different omics reveal varying regulatory patterns for several regulators or metabolites under individual heat or drought stress and combined heat and drought conditions (**Figure 2**), such as the HSPs that are differentially regulated at the transcript, protein and phosphoprotein level as mentioned above (Rizhsky et al., 2002; Rizhsky et al., 2004; Hu et al., 2015; Zhao et al., 2016; Mahalingam et al., 2022; Rahman et al., 2022; Tang et al., 2023). The expression of HSPs and the HSP protein level is higher under combined stress compared to individual stress. However, the phosphorylation of HSPs is up-regulated under drought stress and down-regulated under heat stress, while no significant change in phosphorylation is observed under combined stress.

Breeding stress-tolerant crop varieties under increased temperature and drought is a fundamental way to help deal with climate change and to assure future food security. Understanding the underlying mechanisms of abiotic stress tolerance in crops is crucial to address how abiotic stress affects crop yield and quality effectively, and to provide useful markers and genes for genetic improvement. Selecting the crucial players under combined heat and drought stress, allows us to further understand how plants perceive different stresses and integrate these signals into various tissues and organs, which can be used for targeted breeding to improve plant tolerance under heat and drought stress. Despite the numerous transcriptomes, there have been relatively few studies on post-translational modifications (PTMs), such as phosphorylation, ubiquitination, and SUMOylation, under combined heat and drought stress. PTMs play an important role as rapid and reversible molecular switches, effectively regulating biological pathways and processes within cells (Blazek et al., 2015; Coll-Martínez and Crosas, 2019; Xu et al., 2019), and a comprehensive understanding of protein modification under combined stress is thus crucial to fully capture signalling mechanisms. Furthermore, the integration of other omics data, such as the translatome, and the integration of these multiple omics data is the next key step (Li et al., 2022; Tang et al., 2023).

Finally, we advocate for more omics studies under combined stress conditions, focusing on different species and different organs. In order to investigate plant responses under combined stress, it is crucial to consider the intensity, duration, and timing of the stress conditions. Optimal wet lab experimental setups should incorporate representative climate data to ensure accuracy. Additionally, studying responses directly in the field allows for the consideration of other environmental variables, providing more comprehensive results.

## Author contributions

XX wrote the manuscript and generated **Figure 2**. CF generated **Figure 1**. All authors contributed to the article and approved the submitted version.
